# Comparison of Penalized Cox Regression Methods in Low-Dimensional Data with Few-Events: An Application to Dialysis Patients' Data

**Published:** 2019-07-15

**Authors:** Shideh Rafati, Mohammad Reza Baneshi, Laleh Hassani, Abbas Bahrampour

**Affiliations:** ^1^Department of Biostatistics and Epidemiology, Kerman University of Medical Sciences, Kerman, Iran; ^2^Modeling in Health Research Center, Institute for Futures Studies in Health, Kerman University of Medical Sciences, Kerman, Iran; ^3^Mother and Child Welfare Research Center, Hormozgan University of Medical Sciences, Bandar Abbas, Iran.

**Keywords:** Chronic renal failure, Dialysis, Survival, Cox models

## Abstract

**Background:** Dialysis is a dominant therapeutic method in patients with chronic renal failure. The ratio of those who experienced the event to the predictor variables is expressed as event per variable (EPV). When EPV is low, one of the common techniques which may help to manage the problem is penalized Cox regression model (PCRM). The aim of this study was to determine the survival of dialysis patients using the PCRM in low-dimensional data with few events.

**Study design:** A cross-sectional study.

**Methods:** Information of 252 dialysis patients of Bandar Abbas hospitals, southern Iran, from 2010-16 were used. To deal with few mortality cases in the sample, the PCRM (lasso, ridge and elastic net, adaptive lasso) were applied. Models were compared in terms of calibration and discrimination.

**Results:** Thirty-five (13.9%) mortality cases were observed. Dialysis data simulations revealed that the lasso had higher prediction accuracy than other models. For one unit of increase in the level of education, the risk of mortality was reduced by 0.32 (HR=0.68). The risk of mortality was 0.26 (HR=1.26) higher for the unemployed than the employed cases. Other significant factors were the duration of each dialysis session, number of dialysis sessions per week and age of dialysis onset (HR=0.93, 0.95 and 1.33).

**Conclusion:** The performance of penalized models, especially the lasso, was satisfying in low-dimensional data with low EPV based on dialysis data simulation and real data, therefore these models are the good choice for managing of this type of data.

## Introduction

The full defect and irreversible reduction in renal function lasting more than three months are called chronic renal failure and its advanced stage, in which survival depends on transplant or dialysis, entitled the end-stage renal disease (ESRD). Chronic renal failure is a disease with an increasing trend in recent years; in the US, the prevalence and incidence of ESRD have been doubled in the past decade^1^. In Iran, the prevalence and incidence of ESRD have increased from 238 in 1,000,000 in 2000 to 357 in 1,000,000 in 2006^2^. Moreover, 48.5% of the patients with ESRD used transplant, 48.5% hemodialysis and 3% peritoneal dialysis. In 2008, approximately 12500 patients with ESRD used hemodialysis in Iran^3^. Based on the statistics of the Center for Transplant and Special Disease Management of the Ministry of Health and Medical Education, the number of registered ESRD patients was 24,000 and 40,000 in 2004 and 2009, respectively^4^. Dialysis is a process for eliminating extra uremic fluids and products from the body, used when the kidneys fail to do so. Of the common kidney replacement therapies, hemodialysis is the prevalent method for many patients with ESRD^5^.

This disease can have a negative effect on the quality of life of patients due to its chronic and debilitating nature and leads to reduced social interactions, depression, frustration, reducing a person's ability to perform independent daily activities of life and ultimately increasing mortality. With regard to the issues and problems that dialysis patients are facing, important to determine the variables that affect survival of these patients.

Usually, the data sets comprised enough sample size (n) and limited number of independent variables (p), that is n>p, called low dimensional data and in classic statistical procedures the Cox regression is the common applicable method in such a data sets. In contrast, high dimensional data refers to the situations where n<p.

Another issue affects performance of regression models is the number of Events (known as effective sample size) Per number of independent Variables (EPV)^6^. The ratio of those experiencing the noted event to the number of predictor variables or, more precisely, the number of parameters, is expressed as EPV and based on the simulated studies, EPV of 10 to 20 has been recommended. When EPV is low, the coefficients of Cox model are not reliable.

In high dimensional data, EPV is always low. Therefore, penalized Cox regression methods (PCRM), including ridge regression^7^, lasso^8^, elastic net^9^ and adaptive lasso^10^ that shrink some regression coefficients towards zero are applicable^11^.

Even in low dimensional data, EPV might be low. For example, assume a data set when n=150, p=15, n_event_=30. Here, n>p and EPV=2. Even in the case of low dimensional data with low EPV, standard Cox model might not be applicable^12^. Few studies have used PCRM for low-dimensional data with few-events^13,14^.

In this study, we aimed to determine the variables that affect the survival of dialysis patients, especially those added to survival of patients by controlling them, including these variables can be mentioned to treatment duration and number of hemodialysis sessions per week.

## Methods

One function of the models is to predict the risk of a future event. Based on the low number of mortality cases (the outcome of interest) in the sample, standard Cox regression models were not appropriate since the estimated regression coefficients might become invalid and the predictive models might have weak reliability. A strategy used in this situation is the penalized regression method (lasso, ridge, elastic net and adaptive lasso), which can help in the cases where the value of EPV is low.

By maximizing the penalized partial log-likelihood function below, the elastic net regression coefficients are found.

$$\frac{2}{n}\text{l}\left(\beta \right)-\text{λ}\left(\text{α}\overset{p}{\underset{J=1}{\sum }}\hat{w_{J}}\left|\beta_{J}\right|+\frac{1}{2}\left(1-\text{α}\overset{p}{\underset{J=1}{\sum }}\beta_{J}^{2}\right)\right)$$

Where β1,β2,…,βp correspond to regression coefficients, which are in fact, the weight given to each variable by the model. W is called known weights vector, which is a vector contains one for all of coefficients the lasso, the ridge, and the elastic net. l(β) is the partial log-likelihood function for the Cox model and λ>0 refers to as the tuning parameter that λ were selected using 10-fold cross-validation. Larger values of λ lead to smaller regression coefficients. The α parameter varying from zero to one in the elastic net is called a hybrid parameter. The coefficients of the lasso model are obtained based on α=1 and for the ridge model, α=0^12^.

Adaptive lasso is a version of the lasso, where it assigns different weights to different coefficients for penalizing the coefficients in the lasso. The adaptive lasso can have the oracle properties; namely, it can identify the right subset of true variables and can have optimal estimation rate, if the weights cleverly are chosen. For this method, $\hat{{\text{w}}_{\text{j}}}=\frac{1}{\left|{\hat{\beta_{j}}}^{\text{*}}\right|}$ where $\hat{\beta }*$ is an initial estimate of the coefficients and usually obtained through the ridge.

Here, the application of the penalized methods is used to a real data example with a low EPV based on the dialysis data. The data were collected through the Dialysis Ward of Bandar Abbas Hospitals, Iran. This project has been approved by the Ethics Committee ofKerman University of Medical Sciences with No. IR.KMU.REC.1397.599.

Of the total patients admitted from 2010 to 2016, the data of 252 patients were recorded in the dialysis ward. Mortality was considered as the event of interest and censored cases included those who were alive at the end of the study, excluded cases and those treated with kidney transplant. The survival time of the patients was calculated by years from the onset of dialysis to the end of the study in 2016.

The data were collected based on a designed checklist including age, sex, education, job, blood type, marital status, smoking, disease leading to dialysis (diabetes, hypertension, renal stones and obstruction, renal cysts and congenital diseases), dialysis duration(hour per session), number of dialysis sessions per week, history of cardiac-respiratory diseases, history of anemia and familial history of chronic renal failure.

Regarding the low frequency in some categories of independent variables, in the end, 17 variables included sex, job (five indicator variables), blood type (three indicator variables), history of smoking, diabetes and hypertension (all binary), education, dialysis duration (hour per session), number of dialysis sessions per week, body mass index (BMI), age of diagnosis (all continuous) are used in penalized models. For this data, there were 17 regression coefficients and 35 events so the EPV was 2.

Data of 252 patients were randomly divided into training and testing set, and this process was repeated 500 times. Penalized models were used for the training set. The mean of concordance index (C-index), calibration slope (CS) and the root mean square error (RMSE) were used to assess and compare the prediction accuracy of the penalized models in the test set.

The value of 0.5 for C-index shows the inability of the model in differentiating patients and one indicates the full ability of the model in this differentiation^13,15^. The best RMSE has a value close to zero and is calculated as follows.

$$RMSE\left(t\right)=\sqrt{\frac{1}{n}\overset{n}{\underset{i=1}{\sum }}{\left(S_{i}\left(t\right)-{\hat{S}}_{i}\left(t\right)\right)}^{2}}$$

Where S_i_(t) and ${\hat{S}}_{i}\left(t\right)$ are true survival probabilities of ***i*** th individual at time t and survival probabilities estimated by the different models, respectively ^12,16^. CS is the slope obtained by fitting a simple linear regression model to $y=\text{log}\left(\frac{1-S_{i}\left(t\right)}{S_{i}\left(t\right)}\right)$ and $x=\text{log}\left(\frac{1-{\hat{S}}_{i}\left(t\right)}{{\hat{S}}_{i}\left(t\right)}\right)$. The ideal value for the CS is one^12^. CS is related to goodness of fit, which relates to the ability of a model to fit a given set of data^17^.

Moreover, to compare the predictive performance of the models using the dataset of dialysis patients, we simulated 500 survival datasets with a sample size of 252 for two EPV scenarios (2 and 5). We generated survival times regarding Cox proportional hazard model and the exponential distribution applied to generate baseline hazard in cox model.

The predictors were independent of each other and the vector of true coefficients was β= (1.06,-0.298, 0.362, -1.249, 1.195, -0.283, 0.385, -0.397, 0.04, -12.72, -0.21, 0.579, -0.242, -0.253, 0.549, -0.601, 0.099). All continuous predictors were generated from normal distribution and all categorical variables were produced from binomial distribution.

All the analyses and simulations were carried out using software R version 3.5.3. The glmnet package was used for fitting PCRM (lasso, ridge, elastic net and adaptive lasso). The predicted survival probabilities and C-index were calculated with c060 and Hmisc packages, respectively.

## Results

Overall, 252 hemodialysis patients were studied, of them, 35 (13.9%) cases faced the event of death and 217 (86.1%) cases were censored. The median follow-up was 10 years. The 10 and 20-year survival rate of these patients were 0.86 and 0.69, respectively.

Over 80% of the patients were illiterate or low literacy. Most of the women were housekeeper (87.1%) and most of the men were unemployed or retired (55.9%). About 64% of the patients did not smoke any form of tobacco. All the patients, except for one, had at least one disease leading to dialysis. No case of infection with HIV was observed in the patients. For 194 (77%) patients, each session of dialysis took 4 hours. Moreover, 171(67.9%) patients used dialysis three times per week. Table 1 has described demographic, clinical, and laboratory characteristics of patients in baseline.

**Table 1:** Patients Characteristics

**Continuous variables**	**Censored**		**Died**	
	**Mean**	**SD**	**Mean**	**SD**
Age (yr)	53.24	17.32	53.39	18.09
Age starting dialysis	42.89	16.71	42.83	19.40
Body Mass Index	23.11	4.28	21.44	3.68
**Categorical variables**	**Number**	**Percent**	**Number**	**Percent**
Blood group O	92	42.4	16	45.7
Blood group A	57	26.3	5	14.3
Blood group B	58	26.7	14	40.0
Blood group AB	10	4.6	0	0.0
Illiterate	70	32.3	16	45.7
Low literacy	102	47.0	15	42.9
Diploma	35	16.1	2	5.7
Collegiate	10	4.6	2	5.7
Males	117	53.9	19	54.3
Married	179	82.5	28	80.0
Tobacco use	76	35.0	15	42.9
Diabetes	111	51.2	23	65.7
Hypertension	134	61.8	18	51.4
Urinary stones and Kidney obstruction	22	10.1	1	2.9
Renal cysts	11	5.1	0	0.0
Pulmonary heart disease	45	20.7	5	14.3
Congenital disease	4	1.8	0	0.0
Glomerulonephritis	16	7.4	2	5.7
History of CRF in the family	22	10.1	2	5.7
Anemia	165	76.0	30	85.7
Receive of erythropoietin	204	94.0	35	100
HCV	8	3.7	0	0.0
HBV	3	1.4	0	0.0

In Table 2, we showed the internal and external performance of the methods using cross-validation. The ridge followed by the lasso and adaptive lasso provided the best discrimination in both train and test set. In terms of calibration, the lasso is the best in train and test set (1.224 and 1.332, respectively).

**Table 2:** assessing the prediction accuracy of the models in ten-year based on real dialysis data (500 iterations)

**Model**	**C-index**	**SE**	**RMSE** ^a^	**SE**	**CS** ^b^	**SE**
**Train set**						
Cox-lasso	0.734	0.046	0.133	0.021	1.224	0.655
Cox-ridge	0.760	0.049	0.135	0.016	1.958	0.391
Cox-elastic net	0.730	0.053	0.135	0.007	1.826	0.261
Cox-adaptive lasso	0.732	0.052	0.088	0.013	1.547	0.436
**Test set**						
Cox-lasso	0.737	0.069	0.158	0.013	1.332	0.286
Cox-ridge	0.762	0.062	0.159	0.018	1.710	0.426
Cox-elastic net	0.731	0.076	0.158	0.014	1.390	0.275
Cox-adaptive lasso	0.734	0.073	0.099	0.020	1.412	0.383

^a^ Root Mean Square Error ^b^ Calibration Slope

For train set, the best RMSE was attained using the adaptive lasso (0.088), followed by the lasso (0.133), while the elastic net and the ridge had a RMSE of 0.135. The best RMSE was achieved in test set by the adaptive lasso (0.099), followed by the lasso and the elastic net (0.158).

In Table 2, after assessing the performance of the methods based on C-index, RMSE, and CS, these methods have acceptable predictive accuracy in train and test set. No one method dominates the others, and they all seem to have specific strengths.

Table 3 shows the results of assesses and compares the prediction accuracy of PCRM based on mean of C-index, RMSE, and CS of simulated datasets with different EPV. For EPV 2 in Table 3, the highest mean of C-index was attained by the lasso and the ridge (0.672), the lasso and the adaptive lasso performed the best for EPV 5 (0.627).

**Table 3:** comparison of the prediction precision of the models in ten-year based on simulated dialysis data

**Model**	**C-index**	**SE**	**RMSE** ^a^	**SE**	**CS** ^b^	**SE**
**EPV=2**						
Cox-lasso	0.672	0.074	0.075	0.012	1.673	0.852
Cox-ridge	0.672	0.085	0.082	0.018	1.858	0.449
Cox-elastic net	0.669	0.063	0.080	0.016	1.724	0.516
Cox-adaptive lasso	0.670	0.037	0.082	0.018	1.845	0.478
**EPV=5**						
Cox-lasso	0.627	0.042	0.075	0.016	1.307	0.186
Cox-ridge	0.626	0.044	0.102	0.019	1.710	0.426
Cox-elastic net	0.626	0.044	0.100	0.017	1.429	0.217
Cox-adaptive lasso	0.627	0.047	0.102	0.018	1.488	0.338

^a^Root Mean Square Error ^b^Calibration Slope

Findings in Table 3 show that the lasso had the highest prediction precision based on RMSE for both EPVs (0.075). For both EPVs, the lasso had the highest prediction precision of other three models based on the CS (1.673 and 1.307, respectively). Generally, the performance of the lasso in the simulations was superior to that of the ridge, the elastic net and the adaptive Lasso.

Findings of the lasso (Table 4) indicate that BMI, the level of education, occupation, dialysis duration in each session, number of dialysis sessions per week and age of dialysis onset were the most important variables in predicting the survival time of patients in this study.

**Table 4:** the most important variables based on lasso

**Variables**	**Coefficient**	**95% CI**	**Hazard** **Ratio**
BMI	-0.107	(-0.182, -0.019)	0.89
Education	-0.376	(-0.424, -0.319)	0.68
Unemployed occupation ^a^	0.234	(0.194, 0.264)	1.26
Dialysis Duration	-0.068	(-0.111, -0.021)	0.93
Number of Dialysis	-0.051	(-0.078, -0.019)	0.95
Age of dialysis onset	0.292	( 0.230, 0.350)	1.33

^c^ Reference is employed

Based on this table, for one unit of increase in BMI and the level of education, the risk of mortality is reduced by 0.11 and 0.32, respectively. The risk of mortality was 0.26 higher for unemployed patients than the employed ones. Another significant factor was the duration of each dialysis session. For one unit of increase in the duration of dialysis session, the risk of mortality was shrunk by 0.07. Moreover, by controlling other factors, increasing one unit in the number of dialysis session per week reduce the risk of death by 0.05. Findings revealed that for one unit of increase in age of dialysis onset, the risk of mortality was increased 0.33 by adjusting the effect of other variables.

Table 5 shows the most important variables based on the lasso, the elastic net and the adaptive lasso in 500 bootstrap samples drawn from the original dataset.

**Table 5:** The most important variables (500 bootstrap samples)

**Variables**	**Lasso**	**Elastic net**	**Adaptive lasso**
Body mass index	√	√	√
Occupation	√	√	√
Education	√	√	
Dialysis duration (hour)	√	√	
Number of dialyses (per week)	√	√	√
Age of dialysis onset	√	√	
Blood group			√

## Discussion

We aimed to determine the survival of dialysis patients using these methods (lasso, ridge, elastic net and adaptive lasso) for low-dimensional data with few events. The application of penalized methods in low-dimensional data with low EPV, although important, has been used less.

Upon assessing and comparing the performance of PCRM (lasso, ridge, elastic net and adaptive lasso) based on RMSE, C-index and CS in simulation data with two low EPV scenarios (EPV 2 or 5), lasso was the better model than the others. This means the lasso had higher prediction precision than the other three models which is consistent the other study. In mentioned study, lasso Cox regression model was used to determine variable selection and constructed a model for predicting mortality in dialysis patients which good accuracy of lasso was confirmed by C-index and CS^18^.

A penalized approach should certainly be considered when survival models are used in low-dimensional, low EPV settings. Particularly, use of either the ridge or the lasso is recommended in situations where the EPV is lower than five^13^.

It is important to avoid classic methods for low dimensional data with few events. The penalized methods can improve calibration and predictive accuracy^14^ that present study confirms previous studies.

Ridge shrunk regression coefficients close to zero, but no coefficient was exactly zero. Therefore, the variable selection was not performed. In fact, this method is useful for resolving the problem of multicollinearity and model prediction^14^. The prediction accuracy of the lasso was higher than the elastic net and the adaptive lasso based simulation data (Table 3). Besides, the lasso is a popular technique for simultaneous estimation and variable selection. Therefore, we focus on and interpret the most important variables in predicting the survival of patients based on the lasso model.

In this study, the significance of the variables of BMI, education level, occupation and dialysis duration (hours) in each session and the number of dialysis sessions per week, age of dialysis onset were confirmed based on lasso and elastic net.

Based on the lasso findings in this study, patients with higher BMI had lower mortality rate (HR=0.89). Past studies like present study have shown that high BMI is protective in these patients^19-21^. Hazard ratio of death, in comparison with younger patients, increases with patient age, consistent with another study^22^.

Results of all the statistical models used here demonstrated the importance of level of education and occupation also were confirmed^23^. For instance, in the present study, for one unit of increase in the level of education the risk of mortality was reduced 0.32 based on the lasso. This finding can be justified by higher adherence of patients with a higher level of education to treatment.

The risk of mortality was higher in unemployed patients, which is justifiable based on undesirable economic situation for these patients. These patients often have poor quality of life. Therefore, by postponing treatment due to its considerable costs, they do not receive treatment in the best possible manner.

In this study, based on the lasso results, for one unit of increase in the duration of dialysis, the risk of death was decreased by 0.07; the importance of this variable was confirmed elsewhere^24^. This finding can be explained as the longer the duration of dialysis, the more enhanced its quality would be. Although healthcare workers in dialysis wards are aware of this point, they do not pay attention to it due to the large number of patients per work shift and inadequate number of dialysis devices per patients.

Based on the results of this study, increasing one unit in the number of dialysis sessions per week decreases the risk of mortality, compatible with some other studies^3,25^. This finding is consistent with our expectations. In other words, if needed, we expect the patient to recover faster by increasing the number of dialysis sessions per week.

No study so far has compared the performance of PCRM to determine the survival of dialysis patients in low-dimensional data with few-events, done in this study for the first time. However, there were limitations too. One limitation is that semi-parametric PCRM were compared here, and no parametric model and the non-parametric were included for the comparisons. It is recommended for future studies to include alternative parametric and non-parametric models for low-dimensional data with few events.

## Conclusion

The performance of penalized models, especially the lasso, was satisfying in low-dimensional data with few-events based on dialysis data simulation and real data, therefore these models are the good choice for managing of low-dimensional data with low EPV.

## Acknowledgements

We thank staff of Dialysis Ward of Shahid Mohammadi, Pediatric, and Persian Gulf Hospitals, Bandar Abbas, Iran for assist and allow us to collect data.

## Conflict of interest

The authors declare that there is no conflict of interests.

## Funding

No funding was received.

## Highlights

- The lowest RMSE and the best calibration slope are achieved by the lasso, followed by the elastic net.
- For EPV 2, the C-index the lasso and the ridge are equal. For EPV 5, the highest C-index is attained using lasso and adaptive lasso.
- The performance of penalized models, especially the lasso, is satisfying in low-dimensional data with low EPV.
- The most important factors in predicting the survival of dialysis patients are BMI, education, occupation, dialysis duration, dialysis sessions and dialysis onset.
